# Supplementary material for: Topology of synaptic connectivity constrains neuronal stimulus representation, predicting two complementary coding strategies
Source: PLoS One. 2022 Jan 12;17(1):e0261702. doi: 10.1371/journal.pone.0261702 (PMC8754339; doi:10.1371/journal.pone.0261702)
Supplement: S1 File — (PDF) [file pone.0261702.s001.pdf]

Topology of synaptic connectivity constrains neuronal stimulus representation, predicting two complementary coding strategies

Michael W. Reimann, Henri Riihimäki, Jason P. Smith, Jānis Lazovskis, Christoph Pokorny, Ran Levi

Supplementary material

Supplementary explanations

Here we expand on the topological methods presented in Section Topological methods and subsequent sections, and describe the parameters from Table 1 in a more heuristic manner trying to motivate them from a neuroscientific point of view. Suppl. Fig S1 is a visualization of some of the parameters.

In-degree and out-degree

In signal-flow networks, a high in-degree indicates that a vertex has a potential to receive inputs from many other vertices, while high out-degree indicates potentiality to transmit outputs to many vertices. In a neuroscience context, high in-degree of a vertex v indicates that the corresponding neuron is potentially receiving spikes from many neurons, while a high out-degree means the neuron is potentially transmitting its spikes to a large number of synaptically connected neighbors.

Clustering coefficient

The clustering coefficient at a vertex v is a measure of how interconnected the vertices of the neighborhood of v are. A "possible" directed 3-clique at v is formed by two directed edges containing v and two other distinct vertices. This coefficient aims to measure how far away the neighborhood of v is from complete communication, in a directed way, within all its triplets of neighbors.

Density coefficient

Slightly coarser than the clustering coefficient is the 2nd (unnormalized) density coefficient of v, which is the number of directed 3-cliques that contain v, divided by the degree of v. This value is normalized so that v in a complete directed graph on n vertices has density coefficient 1. The idea is that the larger the density coefficient of v is, the more the vertex participates in "informational exchange", as indicated by the presence of many directed 3-cliques.

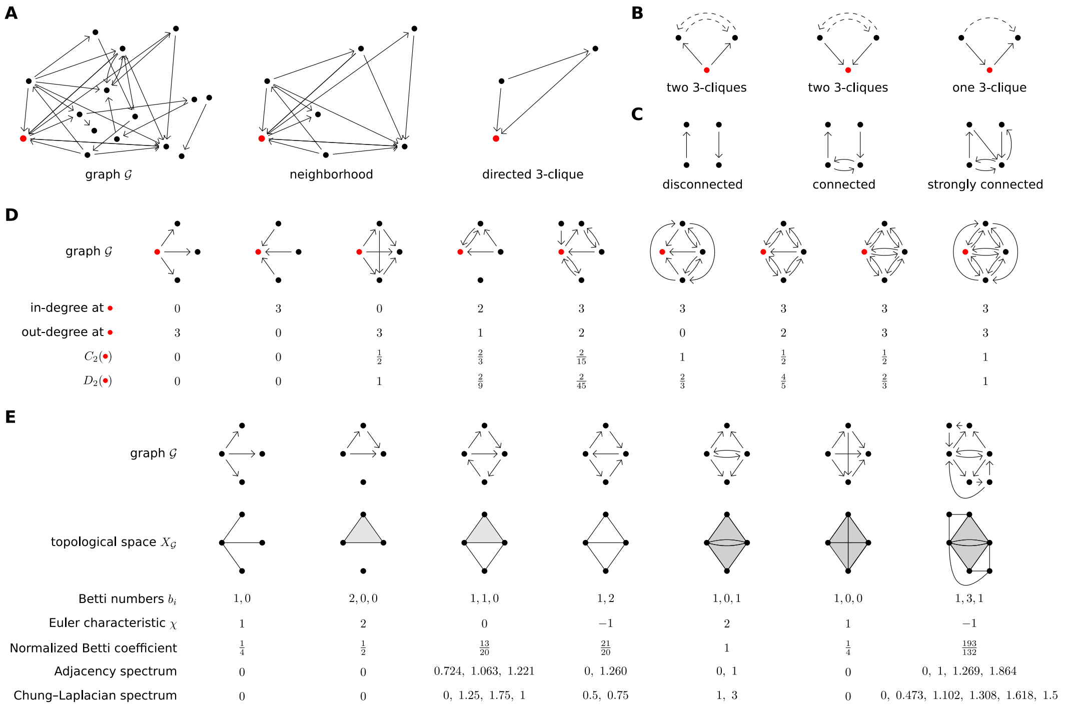

Supplementary Figure S1: A: Graphs, neighborhoods, and cliques. B: Different ways to complete two edges to a directed 3-clique. C: Different types of graphs. Note the Chung–Laplacian spectrum considers only the largest strongly connected component when computing eigenvalues. D: Comparisons of four different graph parameters relative to one of its vertices. E: Comparisons of three different graph parameters and two different spectra (unique absolute values of eigenvalues of matrices).

Homological parameters

The *Betti coefficient* as compared to the Euler characteristic (Section Homological parameters) is a more refined method to summarize the Betti numbers, by adding scaled Betti numbers together. This number is normalized, so that it has value 1 if the topological space X_G associated to the graph G is a sphere (of any dimension). In other words, as the $n+1$ Betti numbers for an n-dimensional sphere are $1, 0, 0, \ldots, 0, 0, 1$, the normalized Betti coefficient measures how close X_G is to being a sphere, where "close" is used in terms of Betti numbers, that is, in terms of different dimensional holes.

Spectral parameters

Solution vectors $\mathbf{x}$ to the characteristic equation $A\mathbf{x} = \lambda\mathbf{x}$ are the eigenvectors of the matrix A. These eigenvectors in the graph setting are vertex functions. When A is the adjacency matrix of a graph, the kth element in the product vector $A\mathbf{x}$ is the sum of the function values at the vertices that the kth vertex has a directed edge to. If this equals the kth element of $\mathbf{x}$ scaled with λ_i as in the characteristic equation, we see that heuristically the eigenvalues of an adjacency

matrix can be seen as scalings of vertex values in a "balanced" signal transmission in a sense that a vertex fully distributes its value to its out-neighbors. The spectral radius and spectral gap (low) then correspond to the maximal and minimal transmission and spectral gap (high) to the difference between the two most maximal transmissions. Similar derivation for Laplacian is not so easy but the Laplacian spectral gap is a measure of robustness of the graph in the sense that how difficult the graph is to disconnect into two separate sets of vertices by removing edges (Section Spectral parameters).

graph $\mathcal{G}$, subset S

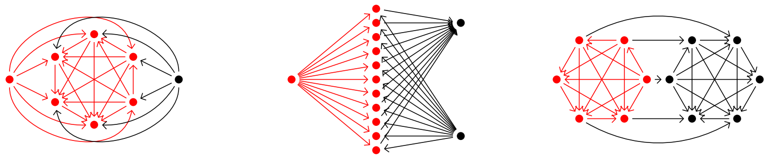

edge boundary ∂S	6	22	5
relative boundary rb(S)	$\frac{6}{21}$	2	$\frac{1}{3}$
afferent extension ae(S)	1	1	0
efferent extension ee(S)	0	1	5

Supplementary Figure S2: Comparisons of the edge boundary, relative boundary, afferent extension, and efferent extension for particular subsets. Subsets are chosen as neighborhoods.

Relative boundary

A subset of vertices in a graph might connect within themselves while also connecting to vertices not in the subset. Relative boundary therefore measures the relative dominance between these two connectivities. It is a measure of the communication potential within a subset, in the same fashion as the clustering coefficient, while also taking into account communication with the ambient graph.

Extension

The afferent extension is defined to measure how many different vertices in the ambient graph might send information into the selected subset of the graph. Likewise the efferent extension measures how much information the subset can potentially send to the ambient graph. Neural spikes flow between neurons through directed synaptic connections and the amount of spikes that can pass through a neuron depends on how many neurons it is connected to. The extension aims to mimic this for a collection of neurons, separating it to incoming and outgoing flow.

Supplementary figures

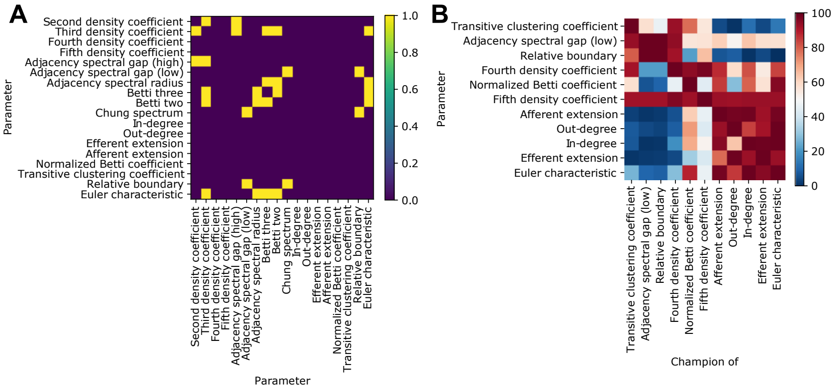

Supplementary Figure S3: A: All investigated topological parameters, with pairs that are mutually redundant (in terms of resulting triad motif expression patterns) highlighted in yellow. B: For the champion neighborhoods of the non-redundant parameters (columns), we consider the values of all parameters (rows), normalized in terms of the percentile of the overall distribution of said parameter (see color bar).

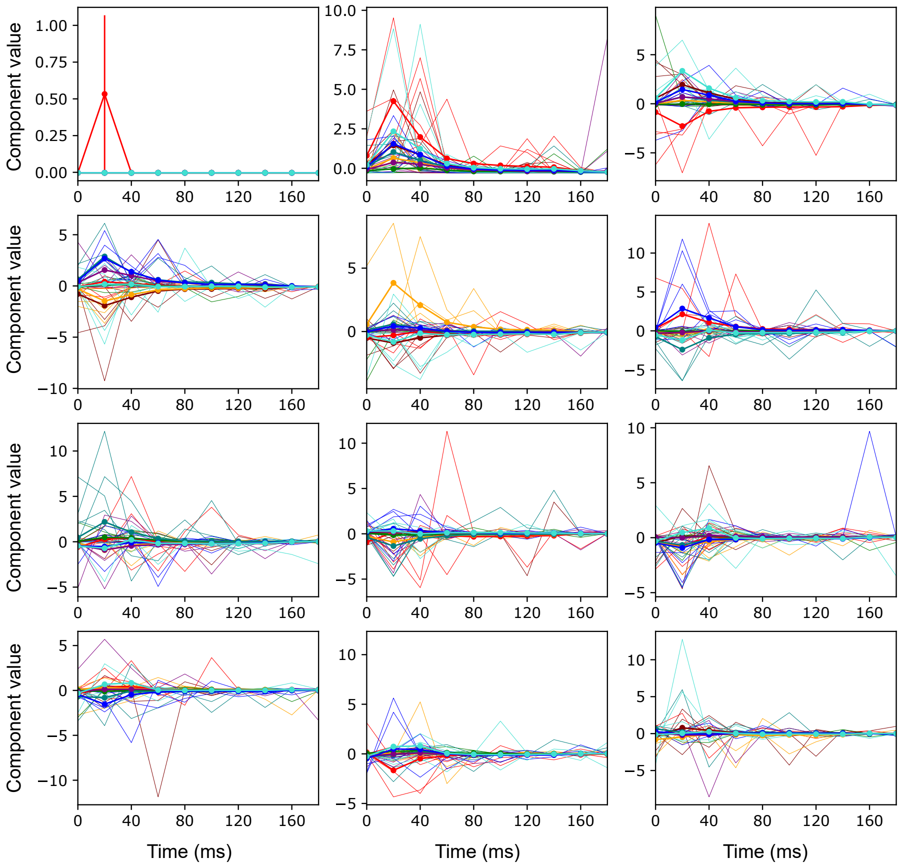

Supplementary Figure S4: Time series of the twelve strongest components (panels) during presentation of the individual stimulus patterns (colored traces). Thick lines and error bars: mean and SEM. Thin lines: for five randomly selected trials using a given pattern.

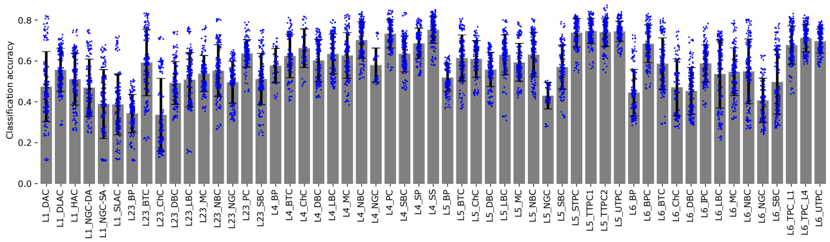

Supplementary Figure S5: Classification accuracies, using the manifold-based method, for the randomly selected neighborhoods with centers of the indicated morphological type. Grey bars and error bars: mean and std. Blue dots: individual neighborhoods.

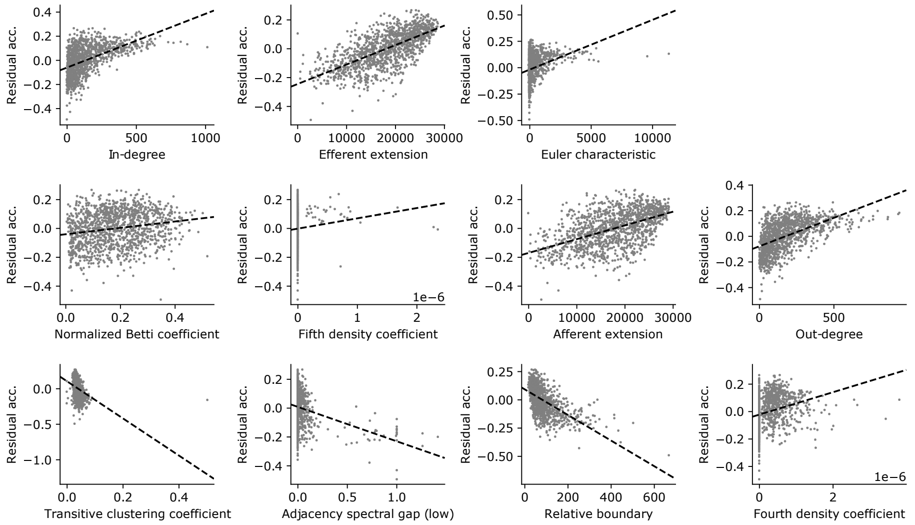

Supplementary Figure S6: Values of topological parameters against residual accuracy for randomly selected neighborhoods. Grey dots: individual neighborhoods. Black line: linear fit.

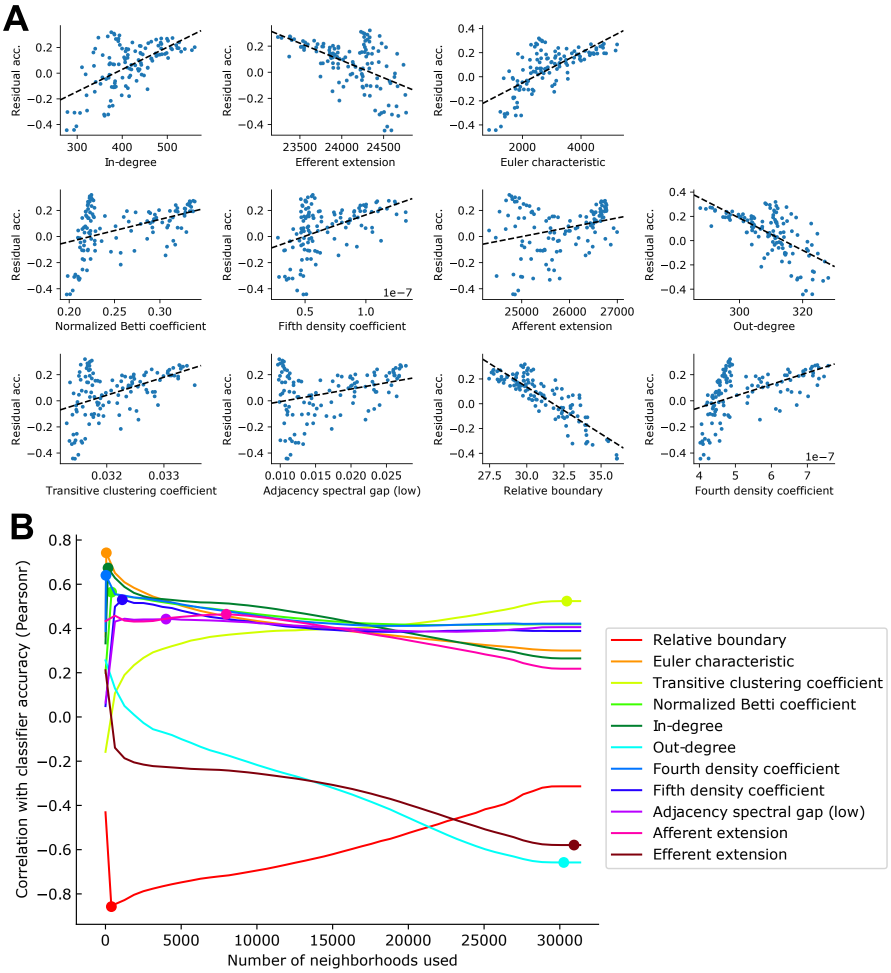

Supplementary Figure S7: A: Synthetic values of topological parameters for volumetric samples against their residual accuracy. Blue dots: individual samples. Black line: linear fit. B: Number of neighborhoods used in the calculation of the synthetic values (see Section Calculating topological parameters for samples) against the resulting correlation (pearsonr) with classifier accuracy. Individual, colored lines: For individual topological parameters. Colored dots: Maxima of the absolute value of correlations, indicating the number of neighborhoods used in the remainder of the manuscript.

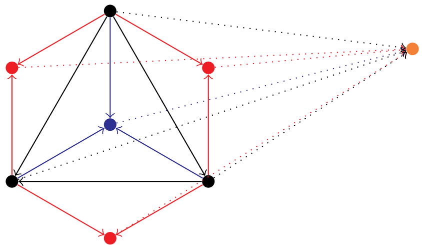

Supplementary Figure S8: The neural circuit to read out the Euler characteristic of the active circuit of a directed 3-clique, the graph in solid black. The red neurons are the read out neurons for the 2-cliques, the blue neuron is the read out clique for the 3-clique. The black and blue dotted lines have weight -1, and the red dotted lines have weight 1. The orange neuron stores the Euler characteristic of the active subgraph.
