# Supplementary material for: Topology of synaptic connectivity constrains neuronal stimulus representation, predicting two complementary coding strategies
Source: PLoS One. 2022 Jan 12;17(1):e0261702. doi: 10.1371/journal.pone.0261702 (PMC8754339; doi:10.1371/journal.pone.0261702)
Supplement: S1 Fig — A: Graphs, neighborhoods, and cliques. B: Different ways to complete two edges to a directed 3-clique. C: Different types of graphs. Note the Chung–Laplacian spectrum considers only the largest strongly connected component when computing eigenvalues. D: Comparisons of four different graph parameters relative to one of its vertices. E: Comparisons of three different graph parameters and two different spectra (unique absolute values of eigenvalues of matrices). (PDF) [file pone.0261702.s002.pdf]

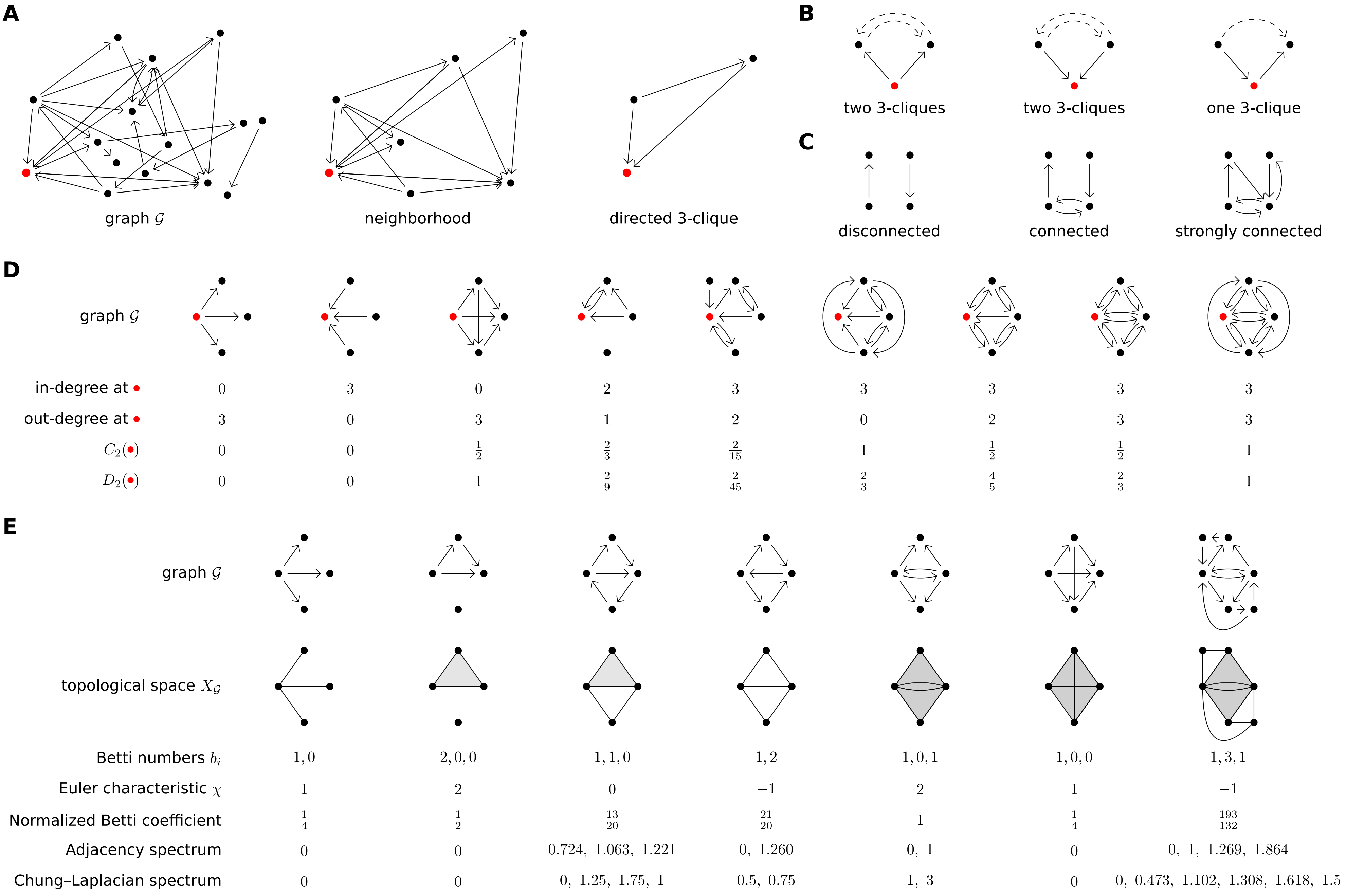
A
graph $\mathcal{G}$
neighborhood
directed 3-clique
B
two 3-cliques
two 3-cliques
one 3-clique
C
disconnected
connected
strongly connected
D
graph $\mathcal{G}$
in-degree at • 0 3 0 2 3 3 3 3 3
out-degree at • 3 0 3 1 2 0 2 3 3
$C_2$(•) 0 0 $\frac{1}{2}$ $\frac{2}{3}$ $\frac{2}{15}$ 1 $\frac{1}{2}$ $\frac{1}{2}$ 1
$D_2$(•) 0 0 1 $\frac{2}{9}$ $\frac{2}{45}$ $\frac{2}{3}$ $\frac{4}{5}$ $\frac{2}{3}$ 1
E
graph $\mathcal{G}$
topological space $X_{\mathcal{G}}$
Betti numbers $b_i$ 1, 0 2, 0, 0 1, 1, 0 1, 2 1, 0, 1 1, 0, 0 1, 3, 1
Euler characteristic $\chi$ 1 2 0 −1 2 1 −1
Normalized Betti coefficient $\frac{1}{4}$ $\frac{1}{2}$ $\frac{13}{20}$ $\frac{21}{20}$ 1 $\frac{1}{4}$ $\frac{193}{132}$
Adjacency spectrum 0 0 0.724, 1.063, 1.221 0, 1.260 0, 1 0 0, 1, 1.269, 1.864
Chung–Laplacian spectrum 0 0 0, 1.25, 1.75, 1 0.5, 0.75 1, 3 0 0, 0.473, 1.102, 1.308, 1.618, 1.5
