# Supplementary material for: Topology of synaptic connectivity constrains neuronal stimulus representation, predicting two complementary coding strategies
Source: PLoS One. 2022 Jan 12;17(1):e0261702. doi: 10.1371/journal.pone.0261702 (PMC8754339; doi:10.1371/journal.pone.0261702)
Supplement: S2 Fig — Subsets are chosen as neighborhoods. (PDF) [file pone.0261702.s003.pdf]

graph $\mathcal{G}$, subset S	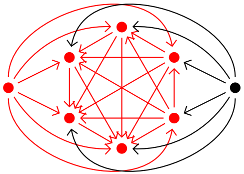	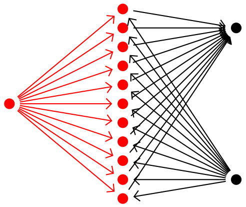	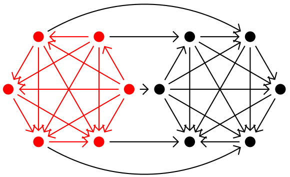
edge boundary ∂S	6	22	5
relative boundary rb(S)	$\frac{6}{21}$	2	$\frac{1}{3}$
afferent extension ae(S)	1	1	0
efferent extension ee(S)	0	1	5
