# Supplementary figures and images for: Topology of synaptic connectivity constrains neuronal stimulus representation, predicting two complementary coding strategies
Source: PLoS One. 2022 Jan 12;17(1):e0261702. doi: 10.1371/journal.pone.0261702 (PMC8754339; doi:10.1371/journal.pone.0261702)

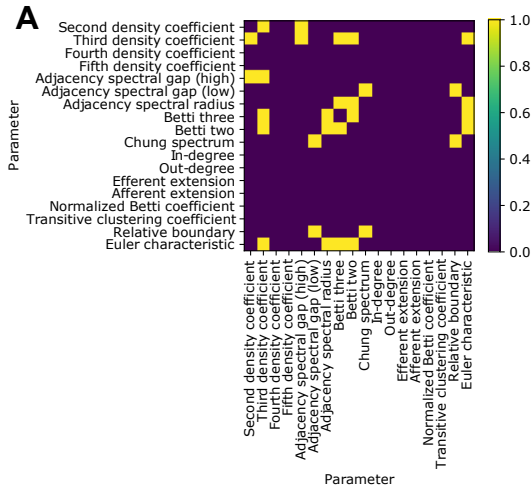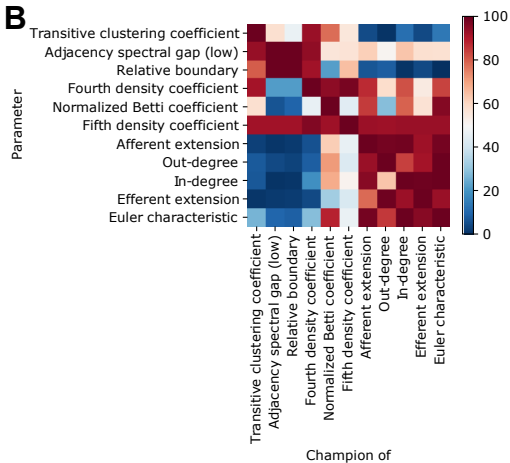

Supplement: S3 Fig — A: All investigated topological parameters, with pairs that are mutually redundant (in terms of resulting triad motif expression patterns) highlighted in yellow. B: For the champion neighborhoods of the non-redundant parameters (columns), we consider the values of all parameters (rows), normalized in terms of the percentile of the overall distribution of said parameter (see color bar). (PDF) [file pone.0261702.s004.pdf]

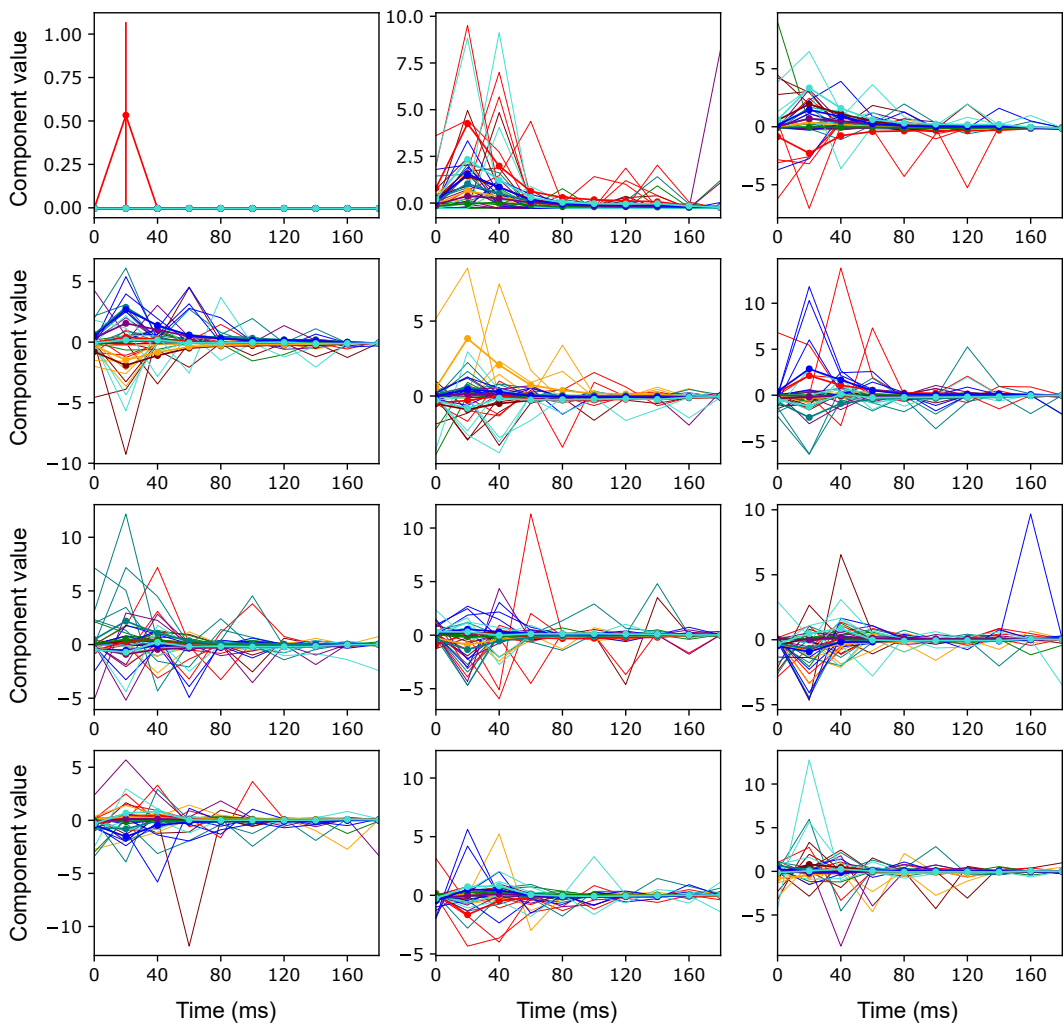

Supplement: S4 Fig — Thick lines and error bars: mean and SEM. Thin lines: for five randomly selected trials using a given pattern. (PDF) [file pone.0261702.s005.pdf]

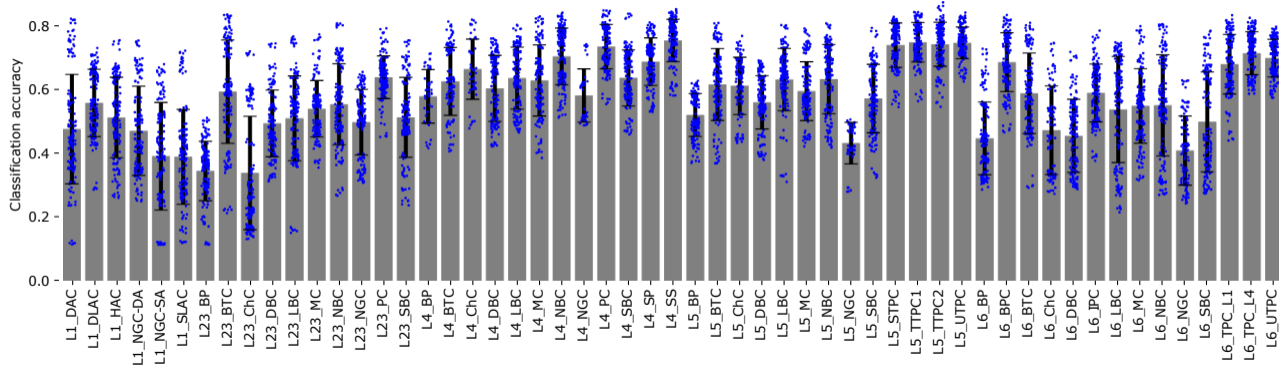

Supplement: S5 Fig — Grey bars and error bars: mean and std. Blue dots: individual neighborhoods. (PDF) [file pone.0261702.s006.pdf]

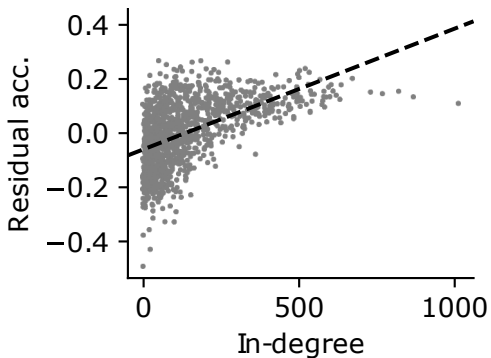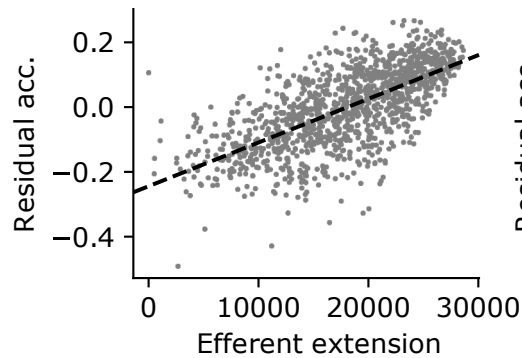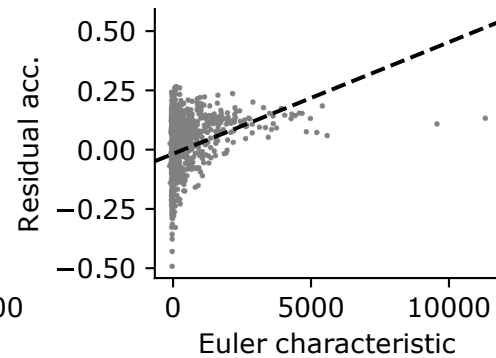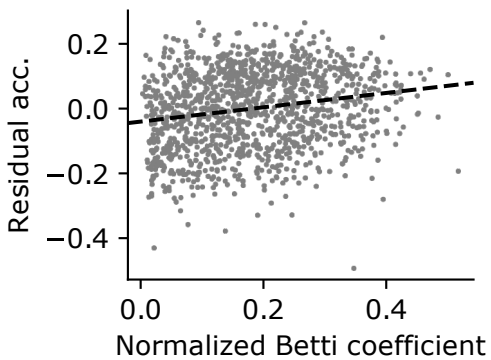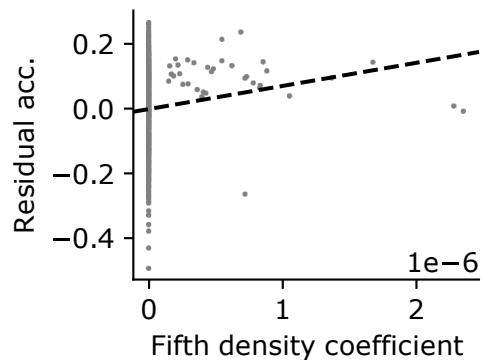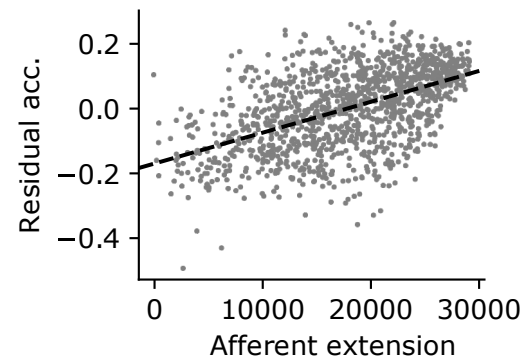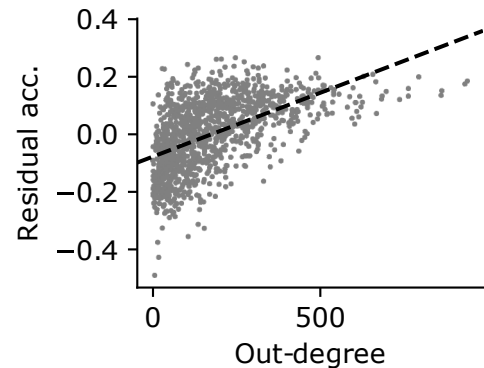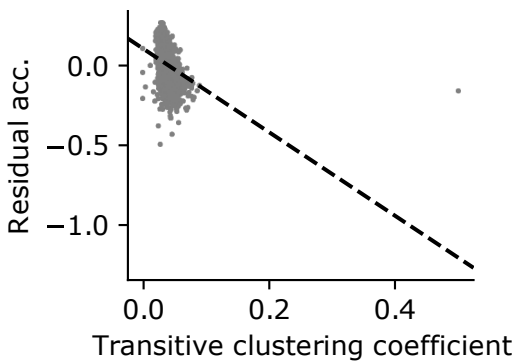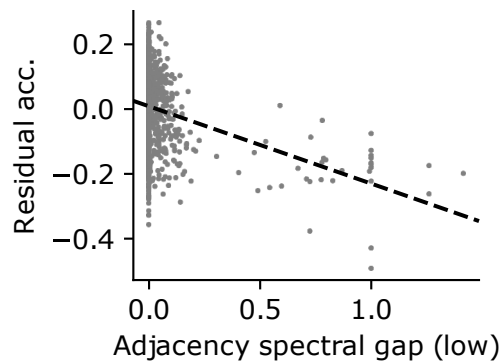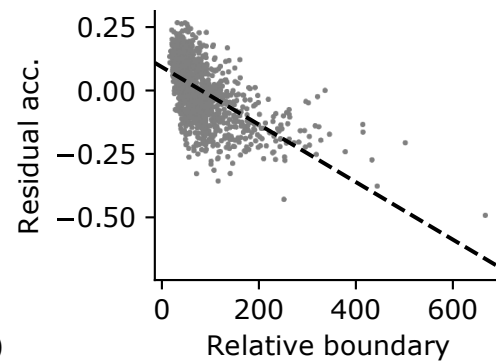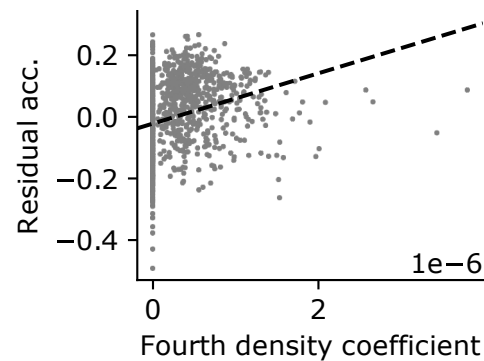

Supplement: S6 Fig — Grey dots: individual neighborhoods. Black line: linear fit. (PDF) [file pone.0261702.s007.pdf]

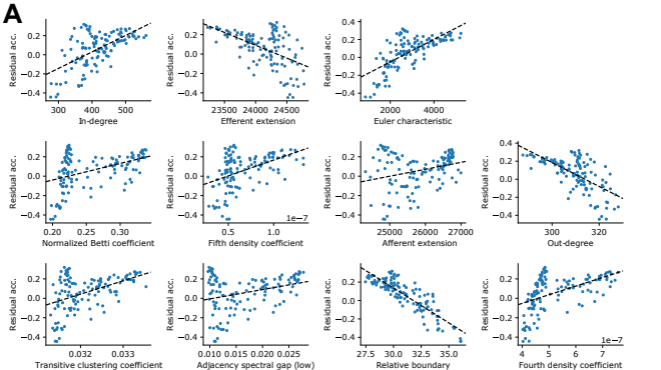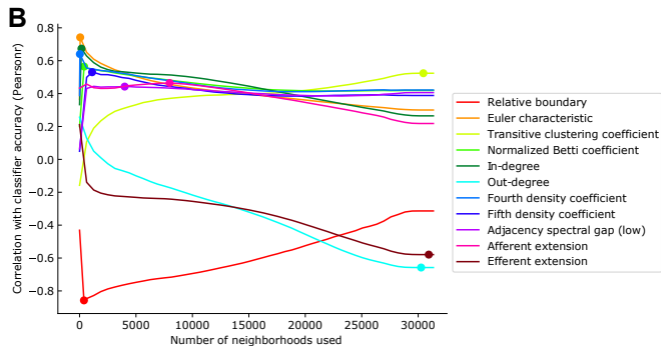

Supplement: S7 Fig — A: Synthetic values of topological parameters for volumetric samples against their residual accuracy. Blue dots: individual samples. Black line: linear fit. B: Number of neighborhoods used in the calculation of the synthetic values (see Section Calculating topological parameters for samples) against the resulting correlation (pearsonr) with classifier accuracy. Individual, colored lines: For individual topological parameters. Colored dots: Maxima of the absolute value of correlations, indicating the number of neighborhoods used in the remainder of the manuscript. (PDF) [file pone.0261702.s008.pdf]
